# Obstetrical Notes and Comments

**Published:** 1910-04

**Authors:** W. T. Marrs

**Affiliations:** Peoria, Ill.


For Texas Medical Journal.

### Obstetrical Notes and Comments.

BY W. T. MARRS, M. D., PEORIA, ILL.

I have before me notes on three hundred obstetrical cases in all phases and conditions of life, from the most indigent, ignorant and illy-environed to the most intelligent, fastidious and well-to-do. My memoranda on these cases have been of much value to me; it is very helpful in after years to turn to your records and read and recall how you extricated yourself from a tight place—oftentimes one that added a few clumps of gray hairs to your (perhaps) now generous shock of "silver locks."

I find that out of this number there were seven pairs of twins; in three cases both babes were girls and all boys in the other three cases. Only in one case were there a boy and a girl. Triplets occurred once, but were premature and did not live.

One woman whom I had attended upon in a confinement developed a phantom pregnancy along about the menopause. While I had never been called upon to make a searching investigation, this woman had manifested all the signs and symptoms of pregnancy throughout its varied cycle—sudden cessation of the menses. vomiting, enlargement of the abdomen, psychic manifestations, etc. I was called at the time of the expected delivery and found the woman in apparent labor; the pains came and went like the real thing. Baby clothes and kindred paraphernalia were in abundance. On examination I found the uterus hard and of normal size, and it struck terror to the heart of this woman to be told that she was looking for a baby that would never come.

In two cases coming under my observation there was no lochia, not a teaspoonful. In each case accommodating old ladies were morally certain that things were "not just right" and, to be candid, I entertained the same opinion. Nevertheless, the important thing in each case is that the women recovered without being sick a moment.

I have learned never to rely absolutely upon any woman's word about matters that concern her organs of generation. A woman may be veracious and dependable on everything else (except possibly her love affairs), but she will prevaricate about the contents of her uterus, and this even when it can be of no earthly use to her to do so. One young woman—already the mother of one child—was delivered of a babe before I arrived on the scene. This woman stoutly asseverated that she did not know that she was pregnant. I have known a number of other women who not only tried to conceal to the very last that they were carrying children, but also denied their condition when questioned by the physician.

Out of this number I find three bastards. Three cases of arrested or incomplete development were observed; two of these were cases of spina bifida and one related to a lack of development of the cranial bones. It is needless to state that all these were attributed to maternal impressions. The funny thing about these impressions is that they always are alleged to occur long after fetal development is past the formative stage. As an illustration: One evening I was at the home of a young woman who expected an early visit from the industrious bird. As the lady was getting out of a carriage she happened to pass near and slightly under one of the horse's head. The old grandmother who was present was quick to discern this movement and reminded the younger woman that she had by this act turned the cord around her baby's neck. In order to undo the mischief thus wrought the pregnant woman was admonished to go back under the horse's neck the other way around and that by so doing she would unwind the cord.

I have always lived in mortal fear of convulsions, although this distressing condition has had little part or parcel in my obstetrical career. I was called once in consultation with another physician to assist in the delivery of a fifteen-year-old girl who was in convulsions. The head of a thirteen-pound baby was resting down well upon the perineum and forcep instrumentation was not difficult. Veratrum viride was given and there was no recurrence of the convulsions.

Breech presentation occurred six times and shoulder presentation twice. If there is anything more difficult than turning a large child in a uterus and other soft parts that are economically built I don't know what it is. It makes one feel like hurling anathemas at the college that crowned him an M. D.

Retained placenta has been the most frequent irregularity that I have met up with, and in several cases the uterus was so firmly contracted for a day or so that curettement was imperative. This condition was not due to the administration of ergot or any other cause that was evident. Most authorities advise letting the after-birth go from thirty minutes to two hours in order that the woman may in a measure recuperate and the uterus regain some of its wonted tone. If there is no apparent danger from hemorrhage or other indications against so doing, I believe in going after the placenta promptly. I have in several cases—not recently—experienced a good deal of trouble with tardy placentas simply because I had been esthetically conservative and let the woman rest until the uterus became in a measure passive. I can see no good physiological reasons for not in most cases delivering the placenta promptly. After the uterus is empty you have a better index on it and can better be able to tell if anything abnormal is going on. And what is still more important, you better know what to do for it.

Post-partum hemorrhage I have found to occur oftenest in anemic and tubercular subjects in whom the hemoglobin element is defective. There are seen occasionally cases in which there is some mechanical impediment which prevents normal uterine contraction. In the latter cases it is justifiable to introduce the hand into the womb and clear out clots and debris; ice may be inserted in the uterus or a stream of very hot or cold water may be thrown into it. Externally the uterus should be squeezed and kneaded with a cold hand. In these desperate cases all fears of the danger from infection sink into desuetude. There may be danger from infection, but it is better to be infected than dead. All hands, instruments, etc., have of course been sterilized at the start of the game. Some epigrammatist has recently said that a doctor and his dignity are soon parted when he meets a case of post-partum hemorrhage; nothing is so calculated to make one get exceedingly busy. Very few women die of post-partum hemorrhage, but the condition is always in a high degree alarming. One sometimes wonders where so much blood can come from. I recall the case of a little anemic woman who bled until a stream ran through the mattress on the floor. She was quite exsanguinated and pulseless, and apparently lifeless. In addition to the measures just mentioned to get the uterus excited to contractions I gave a hypodermic of atropine and glonoin (in some ways antagonistic) and as consciousness returned ergot and brandy were administered. Adrenalin is said to be rather inadequate in these cases, although for such a hemorrhage as metrorrhagia of the menopause it has served me well. It is questionable whether the elevation of the hips is any efficient safeguard against hemorrhage, since it is likely to throw the body in a strained and awkward decupitus and thus possibly hindering normal contractions. The head and shoulders must be low and a snug-fitting band is a *sine qua non.*

Septicemia occurred in three of these cases, one proving fatal. In this fatal case the woman was well on the way to recovery and died suddenly while getting over a bedpan. The immediate cause of death was doubtless embolism. In severe cases the arteries become so impaired and the blood so impoverished and altered in its constituency that anything but a recumbent position is unsafe until vasomotor integrity is well established.

While I have always endeavored to make myself and my operative field clean and sterile, yet I have never been able to trace any connection between filth and infection in the parturient woman. In unsanitary homes where the clothing and person were reeking in filth and perhaps assorted bacteria I have never seen anything but prompt and uneventful recoveries. Let me hasten to remark that I do not say this in an iconoclastic spirit or to in any way disparage aseptic midwifery. But in this class of patients we are not likely to overdo matters in the way of cleansing, douching, etc., thus disturbing nature's protective secretions. It may sound a trifle humorous, but people become accustomed to their own dirt and unkempt environment in consequence of which they become immune to their own brand of germs. Of course this is a very untenable and crude theory, but it seems to work out that way in practice. It is well, however, to teach rigid cleanliness and rational asepsis. If the physician's hands and instruments are sterile he need not worry much about the genital tract.

One of the trying things that confronts the obstetrician when called to many a case of labor is to satisfy himself satisfactory in what manner it is best for him to proceed. It is one of the psychological puzzles of the craft. Considerable experience is necessary in order to satisfy one in a given case, when called early, whether it is best to remain with the woman or to absent oneself and await a return call. During and preceding the first stage the mere presence of the physician often has a deterrent effect upon the progress of the labor. When in doubt as to the time when labor will set in in earnest a sedative can do no harm and may do much good by allaying so-called false pains, thus conducing to strong uterine pains or true labor. If the pains are wholly ineffectual a little morphine, or better codeine, may be given. The woman should not be lulled into a deep or prolonged passivity, but a short nap may change the situation. Caulophyllin is a remedy that has served me well, and it is always safe.

If the condition is such that we feel warranted in encouraging labor we are again often puzzled to know what rational measures and remedies we should resort to. It is hard to maintain a state of "masterly inactivity" and it is bad policy from a psychological standpoint for the physician to sit idly by. There are many methods of facilitating a labor that is a little too slow, and this, too, in a way that is wholly compatible with safety and conservativeness. A procedure that is conducive to good effects in one case may be entirely futile in another. The sitz bath and the upright position are measures that usually produce dilatation and relaxation of the soft parts. Ordinarily I think it best for the woman not to take her bed permanently until the head is engaged. Where there is an excess of amniotic fluid which precludes cephalic engagement an early rupture of the membranes may be justifiable. Usually, however, the "bag of water" serves a most useful purpose, hypostatic dilatation being by many odds preferable to the cephalic wedge. Clearing the lower bowels by the aid of an enema often works wonders and when there is an excess of gas on the stomach an emetic may have a salutary effect. A little relaxation with emetine often gives good results and helps to overcome a rigid os.

I invariably employ sufficient chloroform to blunt the pains, and in nearly all cases it expedites the labor. Its blunting effect upon the pains seems to nerve the woman and enables her to make use of the muscular contractions, or at least better tolerate them and not try to inhibit their expulsive trend. In many cases I have employed one of the Abbott H. M. C. tablets hypodermically, which produces a certain measure of safe anesthesia and only a minimum of chloroform is then required to make the labor practically free from pain. The woman who has enjoyed the beneficient effects of comparative anesthesia in labor will always call for it in subsequent confinements.

This paper is assuming a prodigious length, but before closing

I wish to say a word concerning the oft-heard admonition to the parturient woman to "bear down." The labor process is independent of volition; it is a mechanism that can not be easily explained. The woman should not, therefore, be so much encouraged to "bear down" *per se* as she should be to become non-resistant. She should simply be braced as it were in such a manner as will put the least restraint on the muscles which have the brunt to bear. Labor once thoroughly established will go on in spite of herself and she should be put in such a decupitus as will hamper the process the least.

